# Circular RNA in Acute Central Nervous System Injuries: A New Target for Therapeutic Intervention

**DOI:** 10.3389/fnmol.2022.816182

**Published:** 2022-03-22

**Authors:** Li Zhang, Zhenxing Li, Lei Mao, Handong Wang

**Affiliations:** Department of Neurosurgery, Jinling Hospital, School of Medicine, Nanjing University, Nanjing, China

**Keywords:** acute central nervous system injuries, CircRNAs, cognition function, angiogenesis, apoptosis, downstream molecules

## Abstract

Acute central nervous system (CNS) injuries, including ischemic stroke, traumatic brain injury (TBI), spinal cord injury (SCI) and subarachnoid hemorrhage (SAH), are the most common cause of death and disability around the world. As a kind of non-coding ribonucleic acids (RNAs) with endogenous and conserve, circular RNAs (circRNAs) have recently attracted great attentions due to their functions in diagnosis and treatment of many diseases. A large number of studies have suggested that circRNAs played an important role in brain development and involved in many neurological disorders, particularly in acute CNS injuries. It has been proposed that regulation of circRNAs could improve cognition function, promote angiogenesis, inhibit apoptosis, suppress inflammation, regulate autophagy and protect blood brain barrier (BBB) in acute CNS injuries *via* different molecules and pathways including microRNA (miRNA), nuclear factor kappa-light-chain-enhancer of activated B cells (NF-κB), ph1osphatidylinositol-4,5-bisphosphate 3-kinase/protein kinase B (PI3K/AKT), Notch1 and ten-eleven translocation (TET). Therefore, circRNAs showed great promise as potential targets in acute CNS injuries. In this article, we present a review highlighting the roles of circRNAs in acute CNS injuries. Hence, on the basis of these properties and effects, circRNAs may be developed as therapeutic agents for acute CNS injury patients.

## Introduction

Acute central nervous system (CNS) injuries, including ischemic stroke, traumatic brain injury (TBI), spinal cord injury (SCI), subarachnoid hemorrhage (SAH) and stroke, are neurological emergencies with high risk of neurological decline and death (Caprelli et al., 2019). Acute CNS injuries have a collective global incidence rate of 500–700 per 100,000 people. With high morbidity and mortality around the world, acute CNS injuries usually result in devastating consequences (He et al., 2020). Although some of the pathological processes of acute CNS injuries such as blood brain barrier (BBB) disruption, inflammation and apoptosis have been elucidated, the detailed mechanisms driving these processes are poorly understood (Lee et al., 2019). Despite the progress has been made in the prevention and treatment of acute CNS injuries over the past decades, patients suffering from severe acute CNS injuries usually end up with poor prognosis and require lifelong care, causing substantial financial and emotional cost (Anthony and Couch, 2014). Therefore, it is urgently needed to find optimal therapies and improve patients’ long-term neurological functions after acute CNS injuries.

**FIGURE 1:**
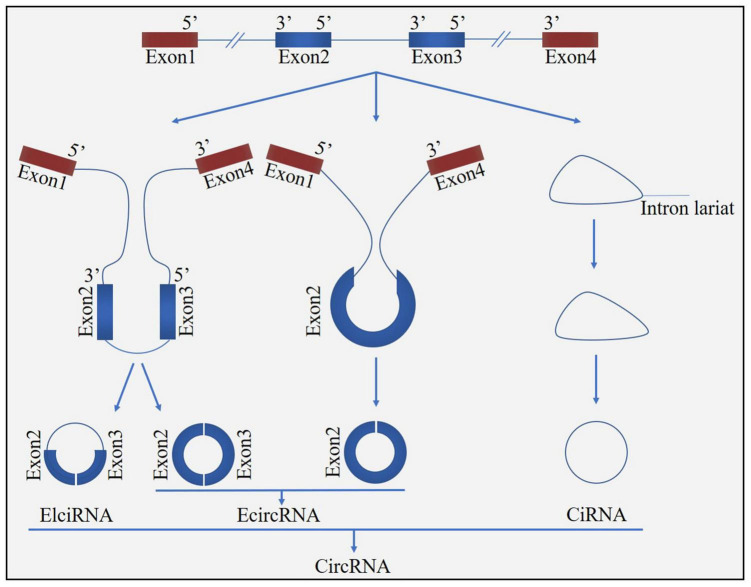
Biogenesis of circRNA. CircRNAs can be formed from either exons (ecircRNAs), introns (ciRNAs), or both exons-introns (elciRNAs).

**FIGURE 2:**
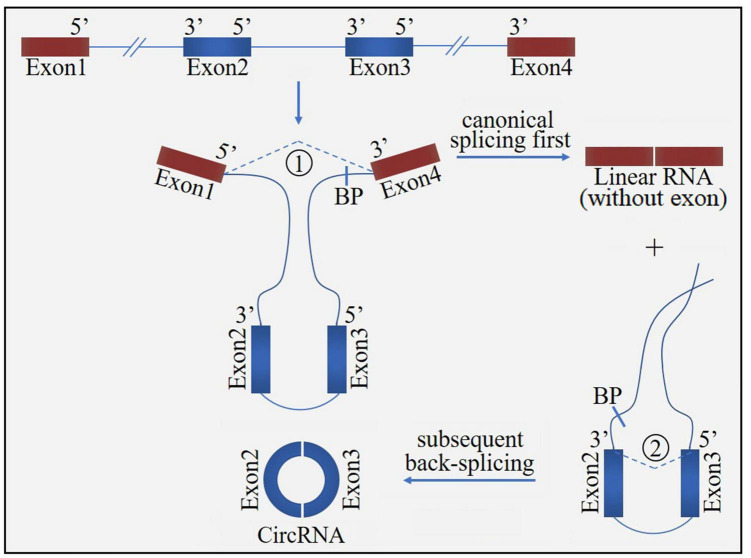
The “exon skipping” or “lariat intermediate” model for circRNA formation. The process starts with canonical splicing for a linear RNA (exon1 and exon4) with skipped exons (exon2 and exon3) and a long intron lariat containing these skipped exons, which is then further back-spliced to form a circRNA. BP, branchpoint.

**FIGURE 3:**
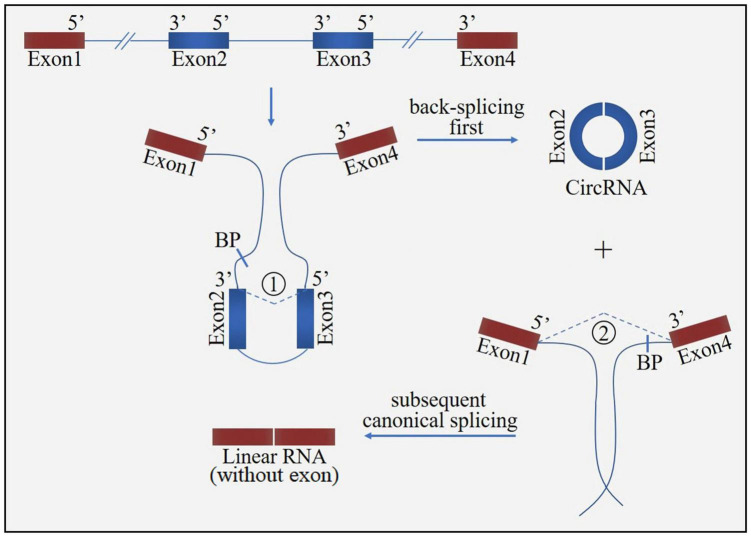
The “direct back-splicing” model for circRNA formation. The process starts with back-splicing for a circRNA (exon2 and exon3) together with an exon-introns-exon intermediate, which further form a linear RNA (exon1 and exon4) with skipped exons. BP, branchpoint.

**FIGURE 4:**
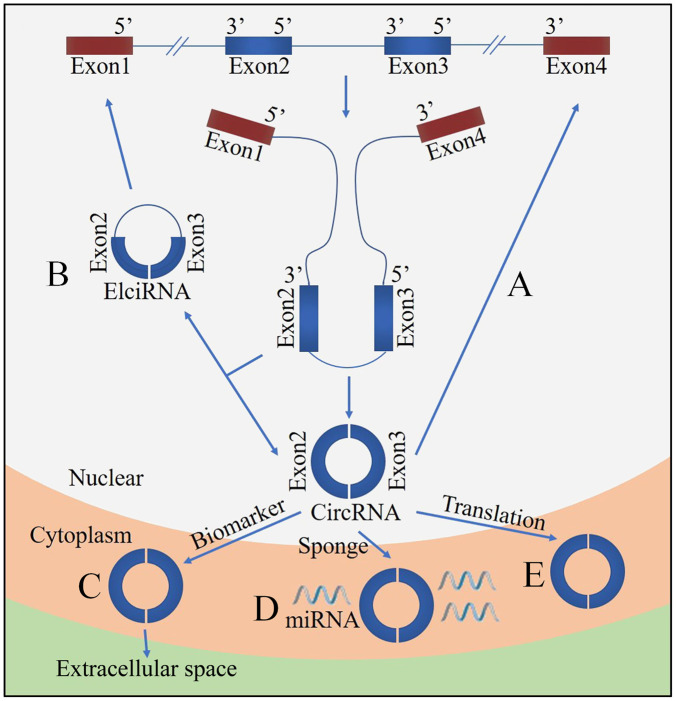
Functions of circRNA. **(A)** CircRNA can compete with the canonical splice of pre-mRNAs. **(B)** Nuclear localized circRNA can affect gene expression via exon intron circRNA (ElciRNA). **(C)** CircRNAs are promising biomarkers. **(D)** CircRNAs can act as sponges of miRNAs. **(E)** CircRNAs can be translated.

Circular ribonucleic acid (circRNA) is a novel class of non-coding RNA generated by pre-mRNA back splicing, which is characterized by a single-stranded, covalently closed-loop structure with no 5′ end caps or 3′ poly (A) tails (Shafabakhsh et al., 2019). CircRNAs are formed from either exons (ecircRNAs), introns (ciRNAs) or both exons-introns (elciRNAs) (Figure 1). The formation of circRNA includes two possible models: the “exon skipping” or “lariat intermediate” model (Figure 2) and the “direct back-splicing” model (Figure 3). The differences between these two models are which step, canonical splicing or back-splicing, happens first (Kristensen et al., 2019). CircRNAs were initially thought to be by-products of transcription with no functions. However, emerging evidences have indicated that circRNAs exhibited biological functions by competing with the canonical splice of pre-mRNAs, affecting gene expression *via* ElciRNA, and acting as transcriptional regulators, microRNA (miR) sponges and biomarkers (Figure 4; Shi et al., 2020b). CircRNAs are expressed in tissue-specific, cell-specific and developmental stage-specific patterns. Due to their specific expression, circRNAs have been implicated in the occurrence and development of several diseases, such as cancers and neurodegenerative diseases (Chen, 2020). In addition, growing studies have identified circRNAs in the pathogenesis of acute CNS injuries, such as ischemic stroke, TBI and SCI. In this regard, circRNAs could be novel therapeutic targets and promising alternative to cell-based therapies for acute CNS injuries, highlighting the potentially roles of circRNAs are important. Herein, we provide an overview of circRNAs functions in acute CNS injuries and the associated molecular mechanisms (Table 1).

**TABLE 1:** The acute central nervous system injuries with the potential role of circular RNAs.

Injuries	CircRNAs	Potential role of circRNAs	Downstream molecules
Stroke	PHKA2	Promote angiogenesis	miR-574-5p/SOD2
	0006768	Attenuate HBMEC injuries	miR-222-3p/VEZF1
	TTC3	Decrease cerebral infarction, brain edema and apoptosis	miR-372-3p/TLR4
	FoxO3	Induce autophagy, attenuate BBB damage	mTORC1
	PHC3	Suppress cell death and apoptosis	miR-455-5p/TRAF3
	DLGAP4	Inhibit cell death and inflammation	miR-503-3p/NEGR1, miR-143
	CDC14A	Relieve infarct volume and astrocytes activation	/
	0025984	Decrease apoptosis and autophagy	miR-143-3p/TET1/ORP150
	016719	Reduce apoptosis	miR-29c/Map2k6
	SKA3	Increase functional outcome	miR-6796-5p/MMP9
	HECTD1	Inhibit apoptosis and inflammation, reduce recurrence	miR-133b/TRAF3, miR-142/TIPARP
	FUNDC1	Improve prediction of stroke associated infection	/
	0072309	Decrease apoptosis	miR-100
	0001599	Biomarker of LAA-stroke diagnosis	/
	CCDC9	Protect BBB, inhibit apoptosis	Notch1
	SHOC2	Suppress neuronal apoptosis	miR-7670-3p/SIRT1
	HIPK2	Increase neuronal plasticity, reduce functional deficits	/
	UCK2	Attenuate cell apoptosis	miR-125b-5p/GDF11
	TLK1	Aggravate neuronal injury and neurological deficits	miR-335-3p/TIPARP
	SCMH1	Promote functional recovery	/
SCI	Usp10	Suppress microglial activation, decrease neuronal death	miRNA-152-5p/CD84
	HIPK3	Alleviate apoptotic injury in neuronal cells	miR-222-3p/DUSP19, miR-588/DPYSL5
	Plek	Inhibit fibrosis activation	miR-135b-5p/TGF-βR1
	Prkcsh	Promote microglia M1 polarization, suppress inflammation	miR-488/MEKK1/JNK/p38
	TYW1	Accelerate neurological recovery	miRNA-380
	2960	Decrease inflammatory response, reduce apoptosis	miRNA-124
	0000962	Attenuate inflammation	miR-302b-3p/PI3K/AKT/NF-κB
	7079	Inhibit apoptosis	/
	0001723	Reduce inflammation	miR-380-3p/NLRP3
TBI	Lrp1b	Inhibit inflammatory response and autophagy	miR-27a-3p/Dram2
	009194	Improve neurological outcomes	miR-145-3p
	Ptpn14	Alleviate ferroptosis and ER stress, protect against BBB damage	miR-351-5p/5-LOX
	chr8_87,859,283-87,904,548	Recover neurological function, decrease inflammation	CXCR2
SAH	ARF3	Attenuate BBB destruction	miR-31-5p/MyD88/NF-κB
	AFF1	Aggravate vascular endothelial cell dysfunction	miR-516b/SAV1/YAP1

*CNS, central nervous system; CircRNAs, circular ribonucleic acids; miRNA, microRNA; PHKA2, phosphorylase kinase alpha subunit; SOD2, superoxide dismutase-2; HBMEC, human brain microvascular endothelial cell; VEZF1, vascular endothelial zinc finger 1; TTC3, tetrapeptide repeat domain 3; TLR4, Toll-like Receptor 4; FoxO3, forkhead box O3; BBB, blood-brain barrier; mTORC1, mechanistic target of rapamycin complex 1; PHC3, polyhomeotic homolog 3; TRAF3, tumor necrosis factor receptor-associated factor 3; NEGR1, neuronal growth regulator 1; CDC14A, cell division cycle 14A; TET1, ten-eleven translocation-1; ORP150, 150-kDa oxygen-regulated protein; Map2k6, mitogen-activated protein kinase 6; SKA3, spindle and kinetochore associated complex subunit 3; MMP9, matrix metalloproteinase-9; HECTD1, HECT domain E3 ubiquitin ligase 1; TIPARP, TCDD-inducible poly-ADP-ribose polymerase; FUNDC1, FUN14 domain-containing 1; LAA, large artery atherosclerosis; SHOC2, Soc-2 suppressor of clear homolog; SIRT1, sirtuin1; HIPK2, homeodomain interacting protein kinase 2; UCK2, uridine-cytidine kinase 2; GDF11, growth differentiation factor 11; TLK1, tousled-like kinases 1; SCMH1, sex comb on midleg homolog-1; SCI, spinal cord injury; Usp10, ubiquitin-specific peptidase 10; CD84, HIPK3, homeodomain-interacting protein kinase 3; DPYSL5, dihydropyrimidinase like 5; Plek, pleckstrin; TGF-βR1, transforming growth factor-beta receptor 1; Prkcsh, protein kinase C substrate 80K-H; MEKK1, mitogen-activated protein kinase kinase 1; JNK, c-Jun N-terminal kinase; PI3K/AKT, phosphatidylinositol-4,5-bisphosphate 3-kinase/protein kinase B; NF-κB, nuclear factor kappa-light-chain-enhancer of activated B cells; NLRP3, NOD-, LRR-, and pyrin-domain containing protein 3; TBI, traumatic brain injury; Lrp1b, lipoprotein receptor-related protein 1b; Dram2, DNA-damage regulated autophagy modulator 2; Ptpn14, protein tyrosine phosphatase non-receptor type 14; ER, endoplasmic reticulum; 5-LOX, 5-lipoxygenase; SAH, subarachnoid hemorrhage; ARF3, ADP-ribosylation factor 3; MyD88, myeloid differentiation factor 88; AFF1, AF4/FMR2 family member 1; SAV1, Salvador 1; YAP1, Yes-associated protein 1.*

## Circular RNAs Expressed in Acute Central Nervous System Injuries

Circular RNAs were firstly reported as viroids in 1976 and firstly detected in human HeLa cells by electron microscopy in 1979. With the development of bioinformatic tools, circRNAs have been found in many species, including unicellular eukaryotes, prokaryotes and mammals (Huang et al., 2020a). Further studies revealed that circRNAs could serve as miRNA sponges or protein scaffolds and being translated into polypeptides (Wang et al., 2018b). These observations led to investigations into the potential role of circRNAs in various models. Recently, the effects of circRNAs in acute CNS injuries were elucidated. Numbers of aberrantly expressed circRNAs were screened out using techniques such as microarray or RNA-sequencing. Specifically, circRNAs such as tousled like kinases 1 (TLK1), discs large-associated protein 4 (DLGAP4), HECT domain E3 ubiquitin ligase 1 (HECTD1), uridine-cytidine kinase 2 (UCK2), AF4/FMR2 family member 1 (AFF1) and SHOC2 were found to affect secondary brain injury in acute CNS injury models (Table 2).

**TABLE 2:** The functions and molecular targets of circRNAs in acute CNS injury models.

CircRNAs	Models	Animals and/or cells	Expression	Beneficial functions of regulation of circRNAs	Molecular targets
TLK1	MCAO	Mice	Increased	Decrease infarct volumes, inhibit neuronal injury, improve neurological deficits	miR-335-3p, TIPARP
DLGAP4	MCAO	Mice	Decreased	Attenuate neurological deficits, decrease infarct areas and BBB damage	miR-143
HECTD1	MCAO	Mice	Increased	Reduce infarct areas, attenuate neuronal deficits, ameliorate astrocyte activation	miR-142
UCK2	OGD/R injury MCAO	HT22 cells Mice	Decreased	Improve neurological deficits, decrease infarct volumes, inhibit neuronal apoptosis	miR-125b-5p, GDF11
SHOC2	OGD/R injury MCAO	Mouse astrocytes Mice	Increased	Decrease cell death and apoptosis, active autophagy	miR-7670-3p, SIRT1
AFF1	Hypoxic injury	Vascular ECs	Increased	Promote the proliferation, tube formation, migration of vascular endothelial cells	miR-516b
SCMH1	PT stroke	Mice, monkeys	Decreased	Improve functional recovery, enhance the neuronal plasticity, inhibit glial activation	MeCP2
Lrp1b	TBI	Rats	Increased	Attenuate neurologic impairment, suppress autophagy and inflammation	miR-27a-3p, Dram2
ANRIL	OGD/R injury	HBMECs	Increased	Inhibit cell damage, apoptosis and inflammation	miR-622
ZNF292	OGD/R injury	H9c2 cells	Increased	Suppress apoptosis and autophagy	BNIP3
CCDC9	MCAO	Mice	Decreased	Protect BBB, inhibit apoptosis	Notch1
TYW1	SCI OGD/R injury	Rats PC12 cells	Decreased	Promote neurological recovery, attenuate apoptosis	miR-380, FGF9
chr8_87,859,283-87,904,548	TBI	Mice	Increased	Recover neurological function, decrease inflammation	CXCR2
0003423	vascular injury	BMECs	Decreased	Increase cell viability, promote angiogenesis, decrease apoptosis	miR-589-5p, TET2
0006768	OGD/R injury	BMECs	Decreased	Accelerate angiogenesis, suppress inflammation	miR-222-3p, VEZF1
7079	SCI	NSC-34 cells	Increased	Reduce apoptosis	/
0025984	OGD/R injury MCAO	Astrocytes Mice	Decreased	Inhibit autophagy and apoptosis	miR-143-3p, TET1
008018	MCAO	Mice	Increased	Attenuate brain tissue damage, neurological deficits and apoptosis	miR-99a, PI3K/AKT
001372	drug-induced brain injury	Rats, PC12 cells	Decreased	Increase cell viability, suppress apoptosis and inflammation	miRNA-148b-3p, PI3K/AKT, NF-κB

*CircRNAs, circular ribonucleic acids; CNS, central nervous system; TLK1, tousled like kinases 1; MCAO, middle cerebral artery occlusion; miRNAs, microRNAs; TIPARP, TCDD inducible poly[ADP-ribose] polymerase; DLGAP4, discs large-associated protein 4; HECTD1, HECT domain E3 ubiquitin ligase 1; UCK2, uridine-cytidine kinase 2; OGD/R, oxygen-glucose deprivation/reoxygenation; GDF11, growth differentiation factor 11; SIRT1, Sirtuin-1; AFF1, AF4/FMR2 family member 1; ECs, endothelial cells; PT, photothrombotic; MeCP2, methyl-CpG binding protein 2; TBI, traumatic brain injury; Dram2, deoxyribonucleic acid damage regulated autophagy modulator 2; HBMECs, human brain microvascular endothelial cells; BNIP3, Bcl-2/adenovirus E1B-19kDa-interacting protein 3; SCI, spinal cord injury; FGF9, fibroblast growth factors 9; CXCR2, C-X-C motif chemokine Receptor 2; BMECs, brain microvascular endothelial cells; TET2, ten-eleven translocation 2; VEZF1, vascular endothelial zinc finger 1; TET1, ten-eleven translocation 1; PI3K/AKT, phosphatidylinositol-4,5-bisphosphate 3-kinase/protein kinase B; NF-κB, nuclear factor kappa-light-chain-enhancer of activated B cells.*

### CircTLK1

CircTLK1, derived from exons 9 and 10 of its host gene TLK1, has the length of 256 nucleotides (nt) and locates on chromosome 2: 171884848–171,902,872 (Song et al., 2020). TLK1 is a known kinase that regulates replication, transcription, deoxyribonucleic acid (DNA) repair, chromatin assembly, chromosome segregation and aggravates cell injury in pathological models, including neuronal injury and myocardial ischemia-reperfusion injury. Knockdown of circTLK1 has no effects on the mRNA and protein levels of TLK1, suggesting that circTLK1 does not encode a protein (Wang et al., 2021). It has been indicated that circTLK1 played an important role in the occurrence and progression of cancer. CircTLK1 could suppress the proliferation and metastasis of cancer cells both *in vitro* and *in vivo* (Lei et al., 2021). However, a growing number of studies have shown that circTLK1 had a special role in recovery after brain injury. It was mainly expressed in neurons and participated in cell proliferation, differentiation and death. CircTLK1 has been shown to exacerbate neuronal injury and neurological deficits after ischemic stroke (Wu et al., 2019).

### CircDLGAP4

CircDLGAP4 is derived from the exons 8–10 of DLGAP4. It is a novel circRNA that participates in the functions of neurons and brain diseases (Bai et al., 2018). DLGAP4 is highly expressed in post-synaptic density (PSD) and is related to the clustering of PSD proteins like PSD95. It is an intrinsic part of the PSD95 core complex. Moreover, DLGAP4 interacts with molecules involved in the N-methyl-D-aspartate receptor signaling pathway in postsynaptic neurons (Feng et al., 2020b). Recently, regulation of circDLGAP4 has been thought to have beneficial effects on neurological disorders. CircDLGAP4 was a strong candidate for early-onset, non-progressive, mild cerebellar ataxia (Chen et al., 2020a). In addition, circDLGAP4 reduced the neurological deficits, infarct areas and BBB damage in models of stroke (Zhu et al., 2019b).

### CircHECTD1

CircHECTD1 is derived from exons 23 and 24 of the HECTD1 gene and promotes functional changes in cells through HECTD1 (Zhou et al., 2018). HECTD1 is an E3 ubiquitin ligase that contains a MIB domain, an N-terminal ankyrin repeat and a C-terminal HECT domain, which plays a vital role in the ubiquitin-proteasome system (Cai et al., 2019). HECTD1 is widely expressed in human and murine primary cells, such as macrophages and neuronal cells, and is required for developmental processes in tissues, including endothelial-mesenchymal transition (EMT), neural tube closure and cell migration (Wang et al., 2020c). It has been shown that HECTD1 regulated neural tube defects in the cranial mesenchyme *via* Hsp90 and cell migration *via* PIPKIγ90 (D’Alonzo et al., 2019). Moreover, previous studies have demonstrated that HECTD1 was involved in regulation of fibrosis and macrophages through ubiquitination. However, it is interesting to find that the effects of HECTD1 on fibroblasts and macrophages were contrary (Bennett et al., 2020). The role of circHECTD1 in acute CNS injuries has also been illustrated. For example, circHECTD1 could decrease infarct areas, attenuate neuronal deficits and ameliorate astrocyte activation in cerebral ischemic stroke (Han et al., 2018).

### CircUCK2

Uridine-cytidine kinase 2, encoded by the UCK2 gene located on chromosome 1q22-23.2, is only expressed in normal human placenta and testis (Yu et al., 2019). UCK2 is a rate-limiting enzyme that phosphorylates uridine and cytidine to the monophosphate form by using adenosine triphosphate (ATP) as the phosphate donor. Besides, UCK2 also catalyzes the phosphorylation of nucleoside analogs, thus playing a significant role in the biosynthesis of pyrimidine nucleotides that synthesize DNA and RNA (Shen et al., 2017). These properties make UCK2 to be an attractive agent of nucleoside prodrugs, such as cyclopentenyl cytidine. Although UCK2 is only expressed in human placenta and testis, upregulation of UCK2 has been observed in many cancers (Malami and Abdul, 2019). Recently, regulation of UCK2 has been proposed in acute CNS injuries. It has been indicated that circUCK2 was significantly downregulated in patients with ischemic stroke and overexpression of circUCK2 attenuated cell apoptosis (Chen et al., 2020b).

### CircAFF1

AF4/FMR2 family member 1 belongs to the AFF (AF4/FMR2) family and participates in the formation of super elongation complex (SEC) by serving as a scaffolding protein. AFF1 contains three conserved domains: an AF4/lymphoid nuclear protein domain, an N-terminal homology domain and a C-terminal homology domain (Chen et al., 2020d). It has been suggested that AFF1 could act as partners of ELL1/2 and positive transcription elongation factor b (P-TEFb) to regulate gene transcription epigenetically and participate in pathological diseases *via* its transcriptional regulatory activity (Kumari et al., 2019). AFF1 has been identified as a related factor of systemic diseases, such as acute lymphoblastic leukemia. Moreover, genome-wide association studies (GWAS) have recognized that mutation of AFF1 was associated with susceptibility to systemic lupus erythematous (Zhou et al., 2017). Recently, the functions of AFF1 in the neural system were investigated. It has been demonstrated that the expression of circAFF1 was upregulated in patients with SAH and overexpression of circAFF1 inhibited the proliferation, tube formation, migration and contributed to apoptosis of endothelial cells (Wang et al., 2020a).

### CircSHOC2

SHOC2 is a scaffold protein that forms the rat sarcoma virus (Ras)-SHOC2-protein phosphatase 1 (PP1) phosphatase complex and positively regulates signaling to extracellular signal-regulated protein kinases 1 and 2 (ERK1/2) (Boned Del Rio et al., 2019). In physiological conditions, SHOC2 plays a pivotal role in regulation of several molecules including activation of transcription factors, crosstalk with the transforming growth factor β (TGF-β) and modulation of genes controlling cell adhesion (Geng et al., 2020). Furthermore, many gain- and loss-of-function approaches found that loss-of-function of SHOC2 was related to impaired cell motility and delays cell attachment, while gain-of-function of SHOC2 was associated with oncogenic ERK signaling in cancer (Silveira et al., 2020). The effects of SHOC2 on brain injuries were also explained. SHOC2 has been shown to promote neuronal autophagy and attenuate neuronal apoptosis *via* the miR-7670-3p/SIRT1 axis in ischemic brain injury (Chen et al., 2020c).

### Other Circular RNAs in Acute Central Nervous System Injuries

Besides these six circRNAs, there were also other circRNAs that have been explored in acute CNS injury models including circSCMH1 (Yang et al., 2020), circLrp1b (Li et al., 2020), circANRIL (Jiang et al., 2020), circZNF292 (Cao et al., 2020), and circCCDC9 (Wu et al., 2020). All these circRNAs played important roles in acute CNS injuries by affecting secondary brain damage.

### Role of Circular RNAs in Acute Central Nervous System Injuries

**FIGURE 5:**
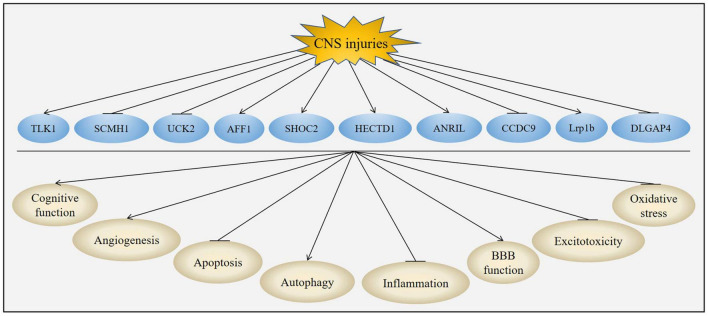
Role of circRNAs in acute CNS injuries. CNS injuries up-regulated the expression of TLK1, AFF1, SHOC2, HECTD1, ANRIL and Lrp1b while down-regulated the expression of SCMH1, UCK2, CCDC9 and DLGAP4. Changes of these circRNAs could improve cognitive function, promote angiogenesis, activate autophagy, protect BBB function, suppress apoptosis, inhibit inflammation, attenuate excitotoxicity and reduce oxidative stress post-CNS injuries.

The unique structures and characteristics of circRNAs make them to be potential candidates for diagnostic biomarkers and therapeutic targets. CircRNA was firstly reported to exhibit neuroprotective effects on acute CNS injuries in Lin et al. (2016). Subsequently, many studies have demonstrated that circRNAs could provide neuroprotective effects in acute CNS injuries. The neuroprotection of circRNAs was attributed to their effects on improvement of cognitive function, inhibition of inflammation, suppression of apoptosis, regulation of autophagy, promotion of angiogenesis, protection of BBB, reduction of excitotoxicity and oxidative stress (Figure 5 and Table 3).

**TABLE 3:** Mechanisms of circRNAs in acute CNS injuries.

Mechanisms	Factors	Associated molecules
Improve cognitive function	Reduce neuronal loss in cortex and hippocampus	/
Attenuate inflammation	Decrease inflammatory factors, cytokines and chemokines	NF-κB, TNF-α, IL-1β, IL-6
Promote angiogenesis	Induce vascular density and endothelial proliferation	VEGF
Inhibit apoptosis	Reduce apoptotic markers and formation of apoptotic bodies	Bcl-2, Bax, caspase-3
Affect autophagy	Increase the expression of LC3 and promote the formation of autophagosome	Beclin-1, LC3
Protect BBB function	Reduce endothelial cell markers and TJ protein loss	GSTα3, GPx
Reduce excitotoxicity	Attenuate the level of glutamate	/
Suppress oxidative stress	Inhibit the level of ROS and RNS	/

*CircRNA, circular ribonucleic acid; CNS, central nervous system; NF-κB, nuclear factor kappa-light-chain-enhancer of activated B cells; TNF-α, tumor necrosis factor-α; IL-1β, interleukin-1β; IL-6, interleukin-6; VEGF, vascular endothelial growth factor; Bcl-2, B-cell lymphoma-2; Bax, Bcl-2-associated X protein; LC3, microtubule-associated protein light chain 3; BBB, blood-brain barrier; TJ, tight junction; GSTα3, glutathione S transferase alpha 3; GPx, glutathione peroxidase; ROS, reactive oxygen species; RNS, reactive nitrogen species.*

### Cognitive Function

Generally, cognitive function is the ability to learn, retain, judge, evaluate and recall information. It includes all mental abilities and processes related to knowledge including memory, reasoning, attention, comprehension and language production (Kennedy, 2019). A variety of chronic diseases such as asthma, cancer, diabetes and heart disease may lead to cognitive decline and result in a disruption of certain cognitive functions such as attention, planning, memory and executive function (Yolland et al., 2020). Cognitive function was originally thought to be regulated by CNS, but now other systems, for example, the immune system and the intestinal microbiome may also be involved (Valentine and Sofuoglu, 2018). Cognitive function impairment may also occur in acute CNS injuries and neurodegenerative disease, which is characterized by problems in thinking, memory, language and social communication. Patients who suffer from cognitive disorder need continuous care from their families and society, increasing the burden of family members and social insurance funds (Birle et al., 2021).

The effects of circRNAs on cognitive function after acute CNS injuries have been explored. In a rat TBI model, knockdown of circLrp1b significant improved spatial learning as measured by the modified Morris water maze test and recovered sensorimotor function as evidenced by decreased mNSS score and brain edema, suggesting that downregulation of circLrp1b could reverse the neurological impairment induced by TBI (Li et al., 2020). Furthermore, overexpression of circUCK2 improved neurological function as evaluated by neurological deficit scoring, adhesive removal test and cylinder test in a mouse cerebral ischemia-reperfusion (I/R) injury model (Chen et al., 2020b).

The precise mechanisms underlying how circRNAs regulated cognitive function were unclear. It has been revealed that cognitive function impairment involved selective neuronal loss in the hippocampus and cortex (Wang et al., 2014). Therefore, circRNAs may also improve cognitive function by intervene with these pathological processes.

### Inflammation

Inflammation plays an essential role in maintaining the homeostasis of tissues under injury, infection or noxious conditions. A typical inflammatory response consists of four components: inflammatory inducers, inflammatory sensors, inflammatory mediators and target tissues (Friedman and Shorey, 2019). Normally, inflammation is related to tissue repair and induces beneficial effects such as phagocytosis of debris and apoptotic cells. However, in respond to CNS injuries, uncontrolled inflammation may result in the production of neurotoxic factors that amplify underlying disease states. This process involves initiating microglia activation and sustaining astrocytic activation (Hori and Kim, 2019). Once activated, these cells can induce a series of events including activation of glial, recruitment of leukocyte, and release of pro-inflammatory cytokines (e.g., interleukin (IL)-1β, tumor necrosis factor-α (TNF-α), interferon γ (IFN-γ)) and chemokines (e.g., C-C motif chemokine ligand 2 (CCL2), C-X-C motif chemokine ligand 8 (CXCL8)). These inflammatory mediators then act on target tissues to induce vasodilation, leakage of plasma and extravasation of neutrophils (Matsuda et al., 2019).

Numerous studies have proposed that circRNAs exerted a central effect in inflammation caused by acute CNS injuries. The effect of circRNAs in acute CNS injuries-induced inflammation was firstly described by Chen et al. (2019). They found that the circchr8_87,859,283-87,904,548 was significantly upregulated in the cerebral cortex around the injury site after TBI. Upregulation of circhr8_87,859,283-87,904,548 further increased the expression of C-X-C motif chemokine receptor 2 (CXCR2) by sponging mmu-let-7a-5p, thus promoting neuro-inflammation and blocking neuronal functional recovery after TBI (Chen et al., 2019). Moreover, in an *in vitro* model of I/R injury, silencing of circANRIL attenuated oxygen-glucose deprivation and reoxygenation (OGD/R)-induced activation of nuclear factor kappa-light-chain-enhancer of activated B cells (NF-κB) and secretion of IL-1β, IL-6, TNF-α, monocyte chemoattractant protein-1 (MCP-1) in human brain microvascular endothelial cells (BMECs), suggesting that circANRIL silencing inhibited the inflammation in I/R injury (Jiang et al., 2020). Furthermore, in a rat TBI model, the production of inflammatory cytokines, including IL-1β, IL-6 and TNF-α, induced by TBI was suppressed by downregulation of circLrp1b, indicating the anti-inflammatory effect of circRNAs (Li et al., 2020).

The underlying mechanisms of circRNAs-mediated inflammation are immensely complicated. Studies have indicated that the NF-κB signaling pathway might be the key target and we will discuss the detailed mechanisms in our following sections.

### Angiogenesis

Angiogenesis is defined as the formation of new blood vessels from pre-existing vessels. It depends on the pro-angiogenic and anti-angiogenic molecules that regulate endothelial cell proliferation and migration (Salehi et al., 2017). Angiogenesis may occur in physiological conditions, such as reproduction, embryonic development and wound healing. Angiogenesis facilitates the generation of new vasculature, which further accelerates highly coupled neurorestorative process and improves tissue perfusion (Zhang et al., 2020). However, abnormal angiogenesis plays a critical role in many pathological processes such as cancer, neurodegenerative diseases and acute CNS injuries (Shim and Madsen, 2018). Angiogenesis is controlled by vascular growth factors such as vascular endothelial growth factor (VEGF). VEGF owns a mitogenic effect on endothelial cells, thus increasing the vascular permeability and promoting cell migration. Besides its role in angiogenesis, VEGF also shows important effects in the neuronal development and physiological function (Melincovici et al., 2018). These features have led to the development of pharmacological agents for anti-angiogenesis by blockade of VEGF signaling.

Since angiogenesis is benefit for acute CNS injuries-induced secondary damage, regulation of circRNAs may attenuate brain injury by promoting angiogenesis. Consistent with this hypothesis, Yu et al. (2021) proposed that in a oxidized low density lipoprotein (ox-LDL)-induced cerebrovascular cell injury model, overexpression of circ0003423 increased the vascular density and angiogenesis as proven by tube formation assay in brain microvascular endothelial cells (BMECs). In another study, it has been shown that upregulation of circ0006768 increased the formation of blood vessels in OGD/R-induced brain injury model, indicating that circRNAs could promote angiogenesis (Li et al., 2021b). Moreover, circRNA.7079, circRNA.7078 and circRNA.6777 were found to play key roles in the vascular endothelial proliferation, migration, and angiogenesis, and may represent therapeutic targets for SCI (Ye et al., 2021). Because angiogenesis is emerging as therapeutic target for acute CNS injuries, therefore, circRNAs-based therapies by targeting angiogenesis might provide opportunities for the development of novel therapeutic strategies for acute CNS injuries.

### Apoptosis

Apoptosis is a very tightly programmed cell death (PCD) occurring regularly to eliminate damaged cells such as those generating following DNA damage or during development (Uzdensky, 2019). Under normal conditions, apoptosis confers advantages to multicellular organisms by maintaining the homeostatic balance between cell survival and cell death (Chi et al., 2018). Apoptosis participates in various physiologic processes such as normal cell turnover, embryonic development and immune system function. However, insufficient apoptosis can trigger cancer or autoimmunity while excessive apoptosis can contribute to abnormal cell death, particularly in pathological conditions such as acute and chronic degenerative diseases, immunodeficiency and trauma (Wang et al., 2020d). Morphologically, apoptosis renders the cell with shrinkage, which is characterized by DNA fragmentation, chromatin condensation, cytoplasm compacting and plasma membrane blebbing. This is followed by nuclear fragmentation and formation of apoptotic bodies (Radak et al., 2017). If apoptosis occurs in acute CNS injuries, it can cause secondary brain injury, aggravating the damage of brain (Bruggeman et al., 2021).

The functions of circRNAs in apoptosis have been studied. The results obtained by Chen et al. (2020b) demonstrated that overexpression of circUCK2 increased cell survival and decreased neuronal apoptosis in cerebral I/R injury as demonstrated by neuronal survival, cell counting kit-8 (CCK-8) assay and TdT-mediated dUTP Nick-End labeling (TUNEL) staining. In addition, Wu et al. (2020) showed that in a mouse ischemic stroke model, overexpression of circCCDC9 upregulated the expression of B-cell lymphoma-2 (Bcl-2) while downregulated the expressions of Bcl-2-associated X protein (Bax) and caspase-3, suggesting that circCCDC9 attenuated cell apoptosis. In another study conducted by Yao et al. (2020) they found that circ7079 decreased apoptosis in neurons following traumatic SCI as measured by Annexin V/propidium iodide (PI) staining and flow cytometry assay, indicating the anti-apoptotic role of circ7079 in neurons. In conclusion, these data suggested that regulation of circRNAs could reduce cell apoptosis in models of acute CNS injury.

Researches so far have just studied the role of circRNAs on apoptosis in general. However, apoptosis can be divided into two pathways: the mitochondria-dependent pathway (the intrinsic pathway) and the death receptor-dependent pathway (the extrinsic pathway). The intrinsic pathway involves the release of cytochrome c, formation of apoptotic body, and activation of caspase-9 and subsequent caspase-3. The release of cytochrome c is positively regulated by the pro-apoptotic Bcl-2 family members such as Bax, Bcl-2 antagonist killer 1 (Bak), Bid and negatively regulated by the anti-apoptotic Bcl-2 family members such as Bcl-2, B-cell lymphoma-extra-large (Bcl-xL). In contrast, the extrinsic pathway is initiated by the binding of TNF ligand to TNF receptor and the binding of Fas ligand to Fas receptor. After binding, the death receptors allow the binding of an initiator caspase-8 or -10 to form death inducing signaling complex (DISC) through its death effector domain (DED). The activation of caspase-8 relays the death signal to an execution caspase to bring about apoptosis (Xu et al., 2019; Obeng, 2021). Thus, which apoptotic pathway is related to the effects of circRNAs in acute CNS injuries-induced apoptosis remains unclear and further studies are needed to clarify it.

### Autophagy

Autophagy is an evolutionarily conserved eukaryotic cellular recycling process. Autophagy plays a crucial role in cell survival and maintenance by degradation of the cytoplasmic organelles, proteins, macromolecules and recycling of the breakdown products (Wang et al., 2018a). Autophagy begins with the formation of a membrane vesicle called the phagophore, which matures into a spherical lipid bilayer vesicle named the autophagosome. The autophagosome then fuses with a lysosome and degrades the contents in autolysosome (Saha et al., 2018). Recent studies have revealed that the dysfunction of autophagy was implicated in acute CNS injuries and extensive activation of autophagy could lead to type II PCD (Zeng et al., 2020). Up to now, the dual role of autophagy in protection or destruction of acute CNS injuries remains controversial. Shi et al. (2020a) found that in cerebral ischemia-reperfusion rats, inhibiting autophagy by sevoflurane attenuated brain damage, demonstrating a detrimental role of autophagy. Conversely, Ahsan et al. (2019) reported that Urolithin A-activated autophagy protected against ischemic neuronal injury by inhibiting endoplasmic reticulum (ER) stress both *in vitro* and *in vivo*, suggesting that autophagy played a beneficial role in stroke.

There were also studies showing that circRNAs could affect autophagy in acute CNS injuries. However, the roles of circRNAs-regulated autophagy in acute CNS injuries, especially in ischemic stroke, were controversial. Zhou et al. (2021) have found that circ0025984 protected astrocytes from OGD-induced apoptosis by suppressing autophagy through the miR-143-3p/ten-eleven translocation 1 (TET1) pathway, suggesting a detrimental role of autophagy in ischemic stroke. Interestingly, in another study conducted by Chen et al. (2020c) they found that circSHOC2 inhibited OGD-induced neuronal apoptosis by promoting autophagy in ischemic brain injury, suggesting a protective role of circSHOC2 and autophagy in ischemic stroke. The discrepancies may be due to the different circRNAs and cell types used in these two studies. Although the ischemic stroke model (OGD) was the same, the circRNA and cells used in Zhou et al. (2021) experiments were circ0025984 and astrocytes, while the circRNA and cells used in Chen et al. (2020c) experiments were circSHOC2 and neurons. In conclusion, by combination with previous studies, we though that depending on different acute CNS injury models, circRNAs and cell types, autophagy and cell death may have inhibitory, additive or even synergistic effects.

### Blood Brain Barrier Function

Blood brain barrier is a highly specialized, semi-permeable physical barrier that formed by the endothelial cells of microvessels (Fu, 2018). BBB locates at the interface between the CNS and the surrounding environment, and protects the CNS from exogenous molecules, toxic side effects of drugs, harmful agents or microorganisms in the blood. In addition, BBB is a dynamic metabolic interface that can bi-directionally regulate the transport of fluids, solutes and cells (Jiang et al., 2018). Structurally, BBB is formed by brain endothelial cells (BECs) with tight junction (TJ). Dysfunction of BBB is a common pathological feature in acute CNS injuries. Several underlying events are involved in BBB destruction, such as disruption of the TJ, breakdown of the BECs and degradation of the extracellular matrix (Langen et al., 2019). In a transient middle cerebral artery occlusion (MCAO) mouse stroke model, Bai et al. (2018) found that circDLGAP4 overexpression significantly inhibited EMT by regulating TJ protein and mesenchymal cell marker expression, resulting in decreased BBB damage. Moreover, Wu et al. (2020) suggested that circCCDC9 alleviated BBB disruption after cerebral I/R injury as evidenced by decreasing the Evans blue dye extravasation and brain water content.

### Excitotoxicity

Excitotoxicity is a phenomenon that describes the injury of neurons due to neurotoxicity in acute CNS insults such as ischemic stroke and trauma (O’Leary and Nichol, 2018). The underlying mechanisms of excitotoxicity include alteration in glutamate and Ca^2+^ metabolism, dysfunction of glutamate transporters and malfunction of glutamate receptors, especially N-methyl-D-aspartic acid receptors (NMDAR) (Binvignat and Olloquequi, 2020). Exacerbated or prolonged activation of glutamate receptors starts a cascade of neurotoxic mechanisms which ultimately lead to cell death (Ladak et al., 2019). In this process, glutamate is the main factor that induces excitotoxic cell damage. Normally, glutamate plays crucial roles in neuronal growth, axon guidance and synaptic plasticity. However, excessive activation of glutamate leads to the imbalance of neuronal Ca^2+^ homeostasis and final excitotoxicity, resulting in mitochondrion destruction, oxidative stress and inflammation (Pregnolato et al., 2019).

The functions of circRNAs in excitotoxicity have also been well established. It has been shown that in GC-2 cells, circMapk1 showed protective effects in antagonizing bisphenol A (BPA)-induced excitotoxicity by sponging miR-214-3p (Li et al., 2019). Besides, caffeic acid phenethyl ester (CAPE), a major active component of propolis, protected HepG2 cells against cadmium chloride (CdCl2)-induced excitotoxicity by suppressing circ0040768-regulated autophagy (Hao et al., 2020). Therefore, circRNAs may also intervene excitotoxicity in acute CNS injuries, however, further studies are needed to verify it.

### Oxidative Stress

Oxidative stress is a result of imbalance between the oxidant compounds and the antioxidant systems (Dumitrescu et al., 2018). Under physiological conditions, oxidant compounds such as reactive oxygen species (ROS) and reactive nitrogen species (RNS) are generated at moderate concentrations and act as second messengers to regulate signal transduction pathways. However, the excessive generation of oxidant compounds due to depletion of the antioxidant system or excitotoxicity leads to the oxidation of biological molecules such as lipids, proteins, and DNA, resulting in oxidative damage in cells, tissues and organs (Khatri et al., 2018). Oxidative stress has been reported in acute CNS injury models and contributed to the secondary brain damage such as brain edema, BBB destruction and apoptosis (Khatri et al., 2018; Orellana-Urzua et al., 2020).

There were also reports indicating that circRNAs could regulate oxidative stress. Wang et al. (2019) suggested that upregulation of circHIPK3 decreased oxidative damage in cardiac microvascular endothelial cells (CMVECs) *via* the miR-29a/insulin-like growth factor 1 (IGF-1) Pathway. Moreover, Feng et al. (2020a) proposed that circPRKCB silencing attenuated oxidative stress levels in intestinal I/R injury *via* the miR-339-5p/p66Shc signaling pathway both *in vivo* and *in vitro*. In addition, HNGF6A inhibited oxidative stress-induced murine osteoblastic cell apoptosis by targeting the circ0001843/miR-214 pathway (Zhu et al., 2020b). So, whether circRNAs could regulate oxidative stress in acute CNS injuries needed to be further studied.

## Mechanism of Circular RNAs in Acute Central Nervous System Injuries

**FIGURE 6:**
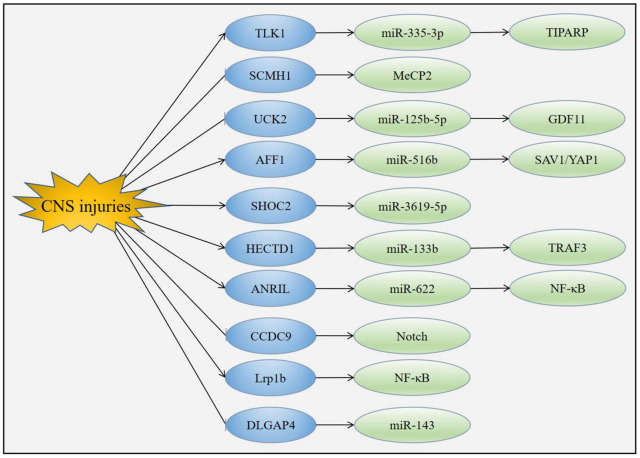
Mechanism of circRNAs in acute CNS injuries. CNS injuries up-regulated the expression of TLK1, AFF1, SHOC2, HECTD1, ANRIL and Lrp1b while down-regulated the expression of SCMH1, UCK2, CCDC9 and DLGAP4. Changes of these circRNAs led to the modulation of downstream molecules such as miRNAs, MeCP2, Notch1 and NF-κB.

The specific mechanisms mediating the functions of circRNAs in acute CNS injuries have yet to be fully explained, a number of downstream molecules of circRNAs have been suggested which may explain their biological effects (Figure 6).

### MicroRNA

MicroRNAs, a subset of non-coding RNAs, are 21 to 23 nucleotides long, single stranded molecules generated endogenously (Tafrihi and Hasheminasab, 2019). MiRNAs are a highly conserved class of tissue specific RNAs with the capability of modulating gene expression by recognition of cognate sequences and interference of transcriptional, translational or epigenetic processes. MiRNAs control a range of physiological and pathological events, including developmental timing, cell proliferation, apoptosis and tumorigenesis (Gjorgjieva et al., 2019). However, illustrating the exact roles of miRNAs in these events are difficult because of their complexity of actions (Saliminejad et al., 2019). Formation of a miRNA consists of two steps, from primary (pri) miRNA to precursor (pre) miRNAs mediated by Drosha in the nucleus and from pre-miRNAs to mature miRNAs mediated by Dicer in the cytoplasm (Mori et al., 2019). As a regulatory element, miRNA itself is also regulated by multiple effectors such as miRNA editing, circadian clock and methylation.

MicroRNAs contribute to nearly 1% of all predicted genes in nematodes, flies and mammals. Because miRNAs influence almost every genetic pathway with a wide range of target genes, an expanding body of evidences have demonstrated that miRNA-based therapies, either activating or inhibiting, hold great promise (Lu and Rothenberg, 2018). Recently, miRNAs have been proposed to be regulated by circRNAs, which played an important role in providing neuroprotection for acute CNS injuries by stimulating angiogenesis, suppressing apoptosis and reducing inflammation. It has been indicated that circTLK1 aggravated neuronal injury and neurological deficits after ischemic stroke *via* miR-335-3p (Wu et al., 2019). Moreover, in human BMECs, knockdown of circANRIL attenuated OGD/R-induced apoptosis and inflammation by sponging miR-622 (Jiang et al., 2020). Furthermore, circDLGAP4 ameliorated neurological deficits, decreased infarct areas, inhibited EMT and maintained BBB integrity in ischemic stroke by targeting miR-143 (Bai et al., 2018).

How circRNAs regulate miRNAs in acute CNS injuries has been explained. CircRNAs contain abundant miRNA response elements (MREs) and can act as competing endogenous RNAs (ceRNAs) by binding to miRNAs, thus regulating the expression of mRNA and affecting the efficiency of miRNAs (Zhu et al., 2019a). In addition, circRNAs may also modulate miRNAs by functioning as miRNAs decoy. These circRNAs are localized in the promoter of their parental genes and associate with RNA polymerase II to enhance the transcription efficiency through the interactions between U1 snRNA/U1A/U1C complex and the 5′-splice site remained in the intron (Hsiao et al., 2017).

### Nuclear Factor Kappa-Light-Chain-Enhancer of Activated B Cells

Nuclear factor kappa-light-chain-enhancer of activated B cells is a family of dimeric transcription factors that has been considered as the central mediator of immune response and inflammatory process (Williams and Gilmore, 2020). NF-κB also regulates the expression of genes that are associated with development, cell proliferation, differentiation, growth, survival and many pathological conditions such as stress responses, apoptosis, inflammation, aging and cancer (Ju Hwang et al., 2019). There are mainly two pathways for NF-κB activation, the “canonical” pathway and the “alternative” pathway. The “canonical” pathway is triggered by microbial products and proinflammatory cytokines such as IL-1 and TNF-α, leading to the activation of RelA- or cRel- containing complexes. The “alternative” pathway is initiated by TNF-family cytokines, B cell activating factor and receptor activator of NF-κB ligand, resulting in activation of RelB/p52 complexes (Jimi et al., 2019).

Nuclear factor kappa-light-chain-enhancer of activated B cells is tightly regulated, and activation of NF-κB has been implicated in acute CNS injuries. CircRNAs were also shown to modulate inflammatory response in acute CNS injuries by activation of NF-κB. In an *in vitro* ischemia-reperfusion injury model, the inflammatory response reflexed by the levels of TNF-α, IL-1β, IL-6 and their mediator, NF-κB were suppressed by silencing of circANRIL (Jiang et al., 2020). Furthermore, in both *in vivo* and *in vitro* ischemic stroke models, downregulation of circHECTD1 inhibited the NF-κB activation in mice MCAO and HT22 cells OGD models, thus inhibiting the inflammatory response (Dai et al., 2021). Therefore, circRNAs were important to regulate inflammation *via* NF-κB in acute CNS injuries.

How circRNAs regulated NF-κB in acute CNS injuries has not been well characterized. It has been reported that the regulation of NF-κB by circRNAs could be mediated by the interaction between circRNAs and the p65 subunit of NF-κB. CircRNA inhibited NF-κB p65 nuclear translocation in a similar manner to IκBα, which formed a circRNA-NF-κB p65 inactive complex by sequence-specific interaction. Besides, circRNA mutants inhibited the interaction of circRNA with NF-κB p65, suggesting that circRNA acted as an endogenous inhibitor of NF-κB signaling (Kong et al., 2019). In addition, miRNAs may be involved in the regulation of NF-κB by circRNAs. MiRNAs could regulate NF-κB by interfere with the signaling components upstream of NF-κB such as affecting the phosphorylation of IKK and IkB. For example, activation of miR-214-3p decreased cell apoptosis and inflammation in osteoarthritis (OA) by downregulated the IKK-β expression and led to the dysfunction of NF-κB signaling pathway (Cao et al., 2021). Since circRNAs can modulate miRNAs by acting as miRNA sponges, we speculate that circRNAs may also regulate NF-κB *via* miRNAs in acute CNS injuries. However, further studies are needed to confirm our hypothesis.

### Phosphatidylinositol-4,5-Bisphosphate 3-Kinase/Protein Kinase B Pathway

The PI3K/AKT is an intracellular signaling pathway that participates in a broad range of cellular processes including proliferation, growth, adhesion, metabolism, metastasis and survival (Huang et al., 2018). This pathway regulates many downstream molecules by collaborating with compensatory signaling pathways, especially the rapidly accelerated fibrosarcoma (Raf)/mitogen-activated protein kinase (MEK)/extracellular signal-regulated kinase (ERK) pathway (Gabbouj et al., 2019). Activation of the PI3K/AKT pathway may occur in loss of phosphatase and tensin homolog (PTEN), mutation of Ras and upregulation of growth factor receptors (GFRs) such as epidermal growth factor receptor (EGFR), receptor tyrosine kinase (RTK) and G-protein-coupled receptors (GPCR) (Rai et al., 2019). These factors firstly activate class I PI3K, then phosphorylate phosphatidylinositol 4,5-biphosphate (PIP2) to form phosphatidylinositol 3,4,5-triphosphate (PIP3) and recruit signaling proteins, including AKT. AKT further functions on several biological molecules, including activating mammalian target of rapamycin (mTOR) (Xu et al., 2020).

Regulation of circRNAs has been found to exhibit protective effects in acute CNS injuries by activation of the PI3K/AKT pathway. It has been revealed that silencing of circ008018 suppressed brain tissue damage and neurological deficits in cerebral I/R. Moreover, the decreased phosphorylation of PI3K/AKT induced by I/R was also reversed by circ008018 knockdown (Yang et al., 2018). Furthermore, Wang et al. (2020b) indicated that circ001372 could reduce propofol-induced neuroinflammation and neural apoptosis in rats and PC12 cells by activating the PI3K/AKT pathway.

But how circRNAs regulated the PI3K/AKT pathway in acute CNS injuries was uncertain. Recently, in many cancer models, it has been proposed that the regulation of the PI3K/AKT pathway by circRNAs might be associated with the miRNA/PTEN pathway. That means, circRNAs firstly controlled miRNAs, which further regulated PTEN and the downstream PI3K/AKT pathway. For example, in esophageal squamous cell carcinoma (ESCC), circLARP4 could act as a sponge for miR-1323 and negatively modulated miR-1323 expression. Besides, miR-1323 bound with PTEN, and PTEN expression was negatively regulated by miR-1323 whereas positively regulated by circLARP4. Furthermore, PTEN deficiency activated the PI3K/AKT pathway. Therefore, circLARP4 sponged miR-1323 and hampered tumorigenesis of ESCC through modulating PTEN/PI3K/AKT pathway (Chen et al., 2020e). In addition, circ0000442 acted as a sponge of miR-148b-3p to suppress breast cancer by downregulation of PTEN to activate the PI3K/AKT signaling pathway (Zhang and Mao, 2021). Therefore, combined with these literatures, we suppose that circRNAs may also regulate the PI3K/AKT pathway *via* miRNAs-PTEN axis in acute CNS injuries. Further studies are needed to explore it.

### Notch1

Notch1 is a class I transmembrane protein that regulates cell-to-cell communication and participates in organ development and intracellular balance (Liang et al., 2019). Notch1 signaling is initiated by the interaction of receptor with its ligands Delta-1 or Jagged-1. This ligand-receptor interaction triggers Notch1 intracellular cytoplasmic domain (NICD) which translocates into the nucleus. There, nuclear Notch1 associates with the transcription factor recombining binding protein suppressor of hairless (RBPJ), activates the expression of target genes and regulates a variety of cellular metabolisms (Gharaibeh et al., 2020). Recent studies have demonstrated that the Notch1 signaling pathway was involved in acute CNS injuries. Blockage of the Notch1 signaling pathway could reduce neuronal cell apoptosis, suppress inflammatory response, promote angiogenesis and improve prognosis in acute CNS injuries (Ribeiro et al., 2021). CircRNAs also facilitated Notch1 to provide neuroprotection in ischemic stroke. Wu et al. (2020) found that overexpression of circCCDC9 attenuated apoptosis in mice following cerebral I/R injury by downregulation of the expression of Notch1.

The underlying mechanisms of how circRNAs regulated Nothch1 in acute CNS injuries were unknow. However, the mechanisms have been clarified in other models. In myocardial infarction, circHipk3 activated Notch1 by increasing N1ICD acetylation, thereby increasing N1ICD stability and preventing its degradation (Si et al., 2020). Besides, circRNAs could regulate Notch1 *via* miRNAs. It has been reported that miR-377-3P mediated the functions of circPDK1 in renal cell carcinoma (RCC) by binding to the 3′ untranslated region (UTR) of Notch1, indicating a critical role of miRNA between circRNA and Notch1 signaling (Huang et al., 2020b). Therefore, circRNAs may also regulate Nothch1 through these processes in acute CNS injuries, which is an interesting aspect worth exploring.

The Notch family consists of four receptors, classified as Notch1, Notch2, Notch3, and Notch4 receptors. Notch1 and Notch2 are expressed widely in tissues throughout development. Notch3 is most abundant in pericytes and vascular smooth muscle, and Notch4 is expressed mainly in endothelium (Engler et al., 2018). The distribution differences make the functions of the Notch receptors variants rather distinct. Notch1 mutation leads to vascular malformations, Notch2 mutation causes vascular and renal defects, Notch3 mutation induces viable and fertile, and Notch4 is dispensable for embryonic development (Zanotti and Canalis, 2016). Since Notch1 can be controlled by circRNAs in ischemic stroke, further studies are needed to clarify whether other Notch receptors such as Notch2 and Notch3 can be regulated by circRNAs in acute CNS injuries.

### Ten-Eleven Translocation

Ten-eleven translocation methyl-cytosine dioxygenases comprise three members, TET1, TET2, and TET3, which are iron^+2^ and α-ketoglutarate (αKG) dependent. TETs have orthologs in almost all multicellular organisms and emerge as a family of epigenetic regulators that are important during development (Yan et al., 2020). Enzymes of TETs can catalyze the hydroxylation of DNA 5-methylcytosine (5mC) into 5-hydroxymethylcytosine (5hmC), and further oxidize 5hmC into 5-formylcytosine (5fC) and 5-carboxycytosine (5caC). Subsequently, 5fC and 5caC are excised by thymine DNA glycosylase (TDG) and replaced with unmodified cytosines through base excision repair (BER), resulting in demethylation (Zhu et al., 2020a). CircRNAs have been shown to regulate TETs in acute CNS injuries. Zhou et al. (2021) suggested that overexpression of circ0025984 ameliorated ischemic stroke injury and protected astrocytes from autophagy and apoptosis through upregulation of TET1. In another study, Yu et al. (2021) implied that circ0003423 alleviated ox-LDL-induced human BMECs injury *via* increasing the expression of TET2. The regulation of TETs by circRNAs may also correlate with miRNAs in a posttranscriptional modification process. For example, there were reports demonstrating that miR-29 could regulate DNA demethylation by direct interaction with TET1 through its 3′-UTR (Roquid et al., 2020).

There are still some differences among the three TETs. Although TETs have same dioxygenase region and C-terminal region, the N-terminal region of these three TETs is different. TET1 and TET3 have a CXXC-type zinc finger domain. TET2 has no CXXC DNA-binding domain, but can interact with a CXXC domain protein. In addition, the expression of TET1 and TET2 is increased in embryonic stem cells (ESCs) while TET3 is increased in oocytes (Li et al., 2021a). Both TET1 and TET2 have been confirmed to be regulated by circRNAS in acute CNS injuries, so whether circRNAs can modulate TET3 in acute CNS injuries is uncertain, further researches are needed to explain it.

## Perspectives and Conclusion

Circular RNAs play essential roles in acute CNS injuries and participate in a number of cellular and molecular processes of acute CNS injuries. In this review, we found that circRNAs were closely associated with carbohydrate, lipid and amino acid metabolisms. By targeting miRNAs or proteins, circRNAs worked as part of the ncRNA regulatory network. In addition, we summarized the functions of circRNAs as well as some downstream moleculars of circRNAs in acute CNS injuries. As molecules that can regulate the pathogenesis of acute CNS injuries through multiple pathways, circRNAs can be used as strategic targets or biomarkers with potential application to the diagnosis and treatment of acute CNS injuries. Furthermore, a better understanding of the regulation of the expression of circRNAs will be instrumental in the development of explicit targeting approaches to treat acute CNS injuries. Thus, circRNAs could be attractive therapeutic targets for developing new therapeutic strategies to achieve better outcomes for patients suffering from acute CNS injuries. Continued discoveries in this field will bring novel insights on circRNAs involved in biological functions and disease progression. Ultimately, circRNAs may hold promise for clinical challenges.

## Author Contributions

LZ finished the original manuscript including figures and tables. LM revised the manuscript. ZL revised the manuscript and provided the funding support. HW conceived the whole work design, played a vital role in manuscript submission, and provided the funding support. All authors contributed to the article and approved the submitted version.

## Conflict of Interest

The authors declare that the research was conducted in the absence of any commercial or financial relationships that could be construed as a potential conflict of interest.

## Publisher’s Note

All claims expressed in this article are solely those of the authors and do not necessarily represent those of their affiliated organizations, or those of the publisher, the editors and the reviewers. Any product that may be evaluated in this article, or claim that may be made by its manufacturer, is not guaranteed or endorsed by the publisher.
